# The gut microbiota of chickens in a commercial farm treated with a *Salmonella* phage cocktail

**DOI:** 10.1038/s41598-021-04679-6

**Published:** 2022-01-19

**Authors:** Viviana Clavijo, Tatiana Morales, Martha Josefina Vives-Flores, Alejandro Reyes Muñoz

**Affiliations:** 1 Grupo de Investigación en Biología Computacional y Ecología Microbiana, Universidad de los Andes, Cra 1 #18A-12, Bogotá, Colombia; 2 Centro de Investigaciones Microbiológicas, Universidad de los Andes, Carrera 1 Este #19A-40, Bogotá, Colombia; 3 Max Planck Tandem Group in Computational Biology, Universidad de los Andes, Carrera 1 Este #19A-40, Bogotá, Colombia; 4 Center for Genome Sciences and Systems Biology, Washington University School of Medicine, Saint Louis, MO 63108 USA

**Keywords:** Bacteriophages, Metagenomics, Microbiome

## Abstract

The microbiota in broiler chicken intestines affects the animals’ health, metabolism, and immunity both positively and negatively. Accordingly, it has a significant impact on animal productivity. Phages, host-specific parasites of bacterial cells, are a promising antimicrobial alternative that selectively target pathogens without disturbing the microbiota. The purpose of this study is to further characterize the commensal microbial community at production scale in broiler chickens treated with a Salmonella phage treatment. We evaluated the cecal microbiota of broilers reared in a commercial farming system where a phage cocktail against Salmonella, SalmoFree was supplied to animals. To do so, two field trials were conducted, incorporating three doses of phages in the broilers’ drinking water. Our results showed that the core microbiome (taxa that were present in more than 50% of samples) contained species that are key to microbiota adaptation in the last stage of the production cycle. Among these, there are some important degraders of complex polysaccharides and producers of short chain fatty acids (SCFA) such as *Eisenbergiella* and *Lachnoclostridium*. The phage cocktail did not affect the normal development of the microbiota’s structure. The addition of the phage cocktail resulted in a significant reduction in *Campylobacter* and an increase in *Butyricimonas*, *Helicobacter* and *Rikenellaceae,* which are common inhabitants in chicken gut with known negative and positive effects on their health and metabolism. Altogether, we consider that these results contribute valuable information to the implementation of large-scale phage therapy technologies.

## Introduction

Microbiota is defined as the complete microbial community, including commensal, symbiotic and pathogenic microorganisms that reside on or within a complex multicellular organism (plants, animals and humans). This microbiota includes bacteria, archaea, fungi, protists and viruses^1^. Knowledge about the importance of the microbiota in human and animal health has grown steadily in the past decade. Early studies focused on cataloguing the microbial species that comprises the human microbiota and its correlation with the health or disease of the host^2–4^. Currently, studies are going beyond the examination of correlations to uncover interconnected relationships between the microbiota, the host and pathogenic bacteria^5–7^. These later studies have clearly established that microbiota and their products are essential not only for gut development, but also for shaping the host’s innate immune system, thereby performing multifactorial impacts on the host’s health^2–4^.

In poultry, the microbiota in broiler chickens’ gastrointestinal tract (GIT) has demonstrated its importance for the host’s health, as it has a positive impact on the immune system, the physiology of the GIT, and the animal’s productivity^8^. Likewise, the microbiota of broilers is involved in reducing and preventing colonization by enteric pathogens by competitive exclusion and the production of bacteriostatic and bactericidal substances^9^. Unbalanced microbiota can therefore induce inflammation, leaky gut, or other gut-related disorders^10,11^. In this context, managing gut health is a key aspect to ensuring optimal development and health in poultry.

The composition of broilers’ microbiota is affected by different factors such as age, diet, genetics, and especially the use of antimicrobials^12^. The fact that the most commonly used antibiotics are broad-spectrum implies that antibiotic therapy causes substantial collateral damage to the host’s microbiota by killing non-targeted and usually beneficial bacteria. This side effect can often lead to dysbiosis, further promoting the emergence of antibiotic resistant bacteria and potentially leading to the horizontal transfer of the corresponding resistance genes^13^. Poultry production systems have used antibiotics extensively, not only for therapeutic purposes, but also as growth promoters. Different studies have shown that indiscriminate use of antibiotics reduces the stability of the microbiota in broilers along with the *Lactobacillus* population in their intestines^14–16^. In a recent study, Danzeisen and colleagues discovered that chickens that did not receive antibiotic supplements had a higher diversity of gene families involved in the degradation of starch, cellulose and hemicellulose, potentially leading to a healthier and more adaptive microbial community. This supports the hypothesis that antibiotic overuse can lead to negative effects on chickens’ health^17^. The observations described above has given rise to a growing interest in the management of infections caused by antibiotic resistant pathogens by selectively targeting the disease-causing bacteria, without disturbing the commensal microbiota of the GIT.

Among the different bacterial pathogens in poultry, *Salmonella* is considered one of the most important food safety problems. This bacterium is a gram-negative, foodborne pathogen that is one of the most common causes of acute gastroenteritis in humans worldwide and is becoming an important public health concern that has a significant economic impact. The main source of human *Salmonella* infections is via consumption of poultry products^18^. Furthermore, although a broad-host-range of *Salmonella* serovars do not produce clinical disease in older birds, some of them can cause gastroenteritis in young chicks^19^. Thus, as well as being a public health threat, *Salmonella* also constitutes an economic problem for producers as it can contribute to a reduced feed intake and, therefore, a reduced growth rate. Indeed, it has been estimated that the broilers’ growth rate can be reduced by as much as 29%^20^.

Controlling *Salmonella* outbreaks is thus a priority given the health impacts and large economic losses it can cause. The pre-harvest stage in poultry is a relevant control point, when it is possible to prevent the introduction of the pathogen into the food chain and consequently reduce the possibility of food poisoning among consumers^21^. However, to date, the most common practice to control the pathogen at this stage is performed with antibiotics, risking the appearance of the aforementioned undesirable side effects.

Phages, as host-specific parasites of bacterial cells, are a promising antimicrobial alternative. Particularly, the use of lytic bacteriophages is an alternative that selectively targets a particular pathogen without disturbing the microbiota^22^. Phage therapy has been reported to have additional advantages such as the modulation of the hosts’ immune system and microbiota, potentially improving host health^23^. Furthermore, phages have been evaluated for animal therapy, prophylaxis and reduction of pathogen loads in food products of animal origin^24^, thus serving as a good alternative for the control of *Salmonella* contamination in poultry. The research on *Salmonella* phages in poultry is not new, with reports including the isolation and characterization of the phages, safety assessment, and effectiveness of selected phages in chicken meat and in chickens in laboratory or controlled environments^25–30^. Although most of these studies have been successful, to date there is only one report on the use of phages targeting *Salmonella* at production scale^31^.

We have previously tested a phage cocktail that selectively targets *Salmonella* strains. The cocktail, called SalmoFree, a previously genomically and phenotypically characterized mixture of six *Salmonella* lytic bacteriophages^31^, is able to control a broad range of *Salmonella* serotypes. The phages present in the cocktail have also been characterized by host range, infection assays, stability in chlorine, transmission electron microscopy, genome sequencing, and a safety assessment in broilers kept in cage batteries^31^. We recently demonstrated the effectiveness of the cocktail in reducing the presence of *Salmonella* on a commercial farm^32^, without affecting the animals or the production parameters, thus demonstrating its innocuity at production scale.

One of the major theoretical advantages of phages over antibiotics is that they do not affect the overall structure of the gut microbial community. However, to date there is no report on the effect of phage treatment in the GIT microbiota of poultry at a production scale. Hence, it is important to characterize the microbial communities and analyze any potential changes induced by SalmoFree phage cocktail administration. In this study the analysis was conducted using *16S rRNA* gene amplicon sequencing of the bacterial communities in samples stored from a recently published experimental set up in a commercial farm^32^. Altogether, this work extends the existing knowledge about the microbiota in broiler chickens under farming conditions and will help to reveal possible effects of phage therapy in this scenario.

## Results

The current study is focused on the description of the cecal microbiota of broilers in a commercial scenario, while under the effect of a *Salmonella* phage cocktail, SalmoFree, incorporated as treatment in the broilers’ drinking water. We used previously collected samples from a recently published experimental set-up, in which we assessed the effectiveness of the phage cocktail in the reduction of *Salmonella* by comparing the presence of *Salmonella* and the production parameters of the two treatment groups (with and without SalmoFree)^31^. The summary of those previously published results are presented in Tables 1 and 2. Unfortunately, given the production scale of the experimental setup, although the phage cocktail was administered under the best possible conditions, The results showed a phage cross-contamination between control and treated houses in the first trial and the presence of phages in trial two before administering the treatments in both groups, this issue hinders possible conclusions regarding the effect of the phage treatment in the cecal microbiota of commercial chickens.

**Table 1:** *Salmonella* reduction throughout the production cycle of trials 1 and 2 for farmhouses treated with and without SalmoFree.

Farmhouse/sampling point*	No of cloacal swaps *Salmonella* positive**										
	Trial I						Trial II				
	1b	1a	2b	2a	3b	3a	1b	1a	2b	2a	3b***
Control farmhouse 1	2	3	3	4	1	3	9	7	5	4	2
Control farmhouse 2	5	5	1	2	1	1	9	10	5	3	1
Phage-treated farmhouse 3	4	2	1	4	0	0	10	10	9	6	3
Phage-treated farmhouse 4	5	3	1	2	0	0	9	8	7	2	0

Data modified from Clavijo et al*.*^32^.

*Nomenclature of the sampling point corresponds to the number of dose (1–3) followed by a letter indicating whether it was taken a day before (b) or after (a) the corresponding dose.

**Total number of swaps sampled: n = 5 in Trial 1 and n = 10 in Trial 2.

***Data for samples taken after dose 3 (3a) in Trial 2 are not shown due to the accidental loss of the samples.

**Table 2:** Phage incidence throughout the production cycle of trials 1 and 2 for farmhouses treated with and without SalmoFree.

Farmhouse/dose day^a^	No. of samples positive for amplification of phages gene^b^							
	Trial I				Trial II			
	1b	1a	3b	3a	1b	1a	3b	3a
Control house no. 1	0	1	3	0	0	0	0	0
Control house no. 2	0	1	1	1	1	1	0	3
Treated house No. 3	0	2	5	3	0	4	1	4
Treated house no. 4	0	1	3	4	3	2	2	4

^a^Nomenclature of the dose day corresponds to the number of the dose followed by a letter indicating whether it was taken a day before (b) or after (a) the corresponding dose.

^b^No. of positive samples for amplification of the phage tail fiber protein gene of phages from the total samples where total sample size per time point is 5.

**Figure 1:**
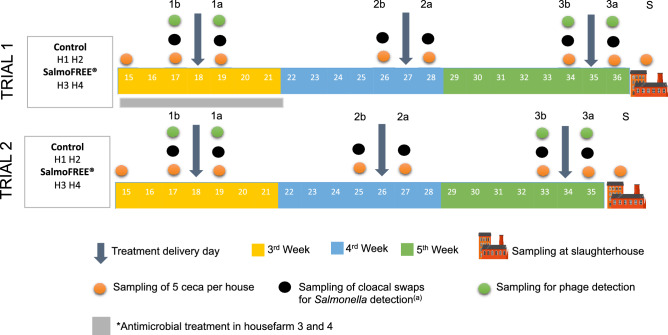
Experimental design and samples taken through the production cycle of trials 1 and 2. The number in the colored boxes corresponds to the cycle day. The color of the boxes corresponds to the week of the cycle (3rd to 5th). The last day corresponds to the slaughter date. Down-pointing arrows indicate the days where the treatment was administered (phage or control). The sampling days are also indicated with colored spheres. The nomenclature of the sampling point corresponds to the dose followed by the letter b (before) or a (after) and at the slaughterhouse (S). The farmhouse labels are H1 and H2 for controls and H3 and H4 for treatments. (a) Total number of individual cloacal swaps sampled were 5 and 10 for Trial 1 and Trial 2 respectively. This figure was made using PowerPoint for Mac v 16.5 (https://www.microsoft.com/en-ww/microsoft-365/mac/microsoft-365-for-mac).

In summary, two independent trials were performed; each one consisting of two treated and two control farmhouses where chickens were followed for the entire growth period up to the slaughterhouse (Fig. 1). Given the commercial setup of the experiment, it prevents the possibility of replicating the trials under controlled conditions. Furthermore, the cost of performing multiple times the proposed experimental setup prevents us from having a statistically significant number of trials. In consequence, the purpose of the two independent trials was to compare and contrast, identifying commonalities and differences. The results obtained from the different farmhouses are not expected to be considered as replicated since true replicates are impossible to achieve in commercial rearing conditions (farmhouses can contain up to 13,400 chickens per house, and the specific conditions are almost impossible to control).

### Sequencing information

The cecal microbiota was characterized at 8 different points in time during the chickens’ growth phase (Fig. 1). The V4 hypervariable region was sequenced using MiSeq v2 2 × 250 pair-end reads. Following quality control, 228 samples were retained, with 4,995,664 sequences and a total of 3993 amplicon sequence variants (ASVs). The count of reads per sample varied from 3527 to 210,082 with an average of 21,911 and a median of 18,130 per sample, which is comparable to previous studies^33,34^.

An initial quality control analysis on raw reads determined that the sequence variants generated for F2 (farmhouse two; control farmhouse) in Trial 2 were significantly different in composition and diversity from the other samples (Fig. S1), likely reflecting a technical rather than a biological effect. Thus, all samples from that farmhouse were excluded from further analysis. Additionally, sequences generated from most of the samples taken one day before the beginning of the growth phase (birds at age 15 and 14 days for the first and second trial, respectively) had a very low yield of DNA extraction leading to a high frequency of failed amplifications and sequencing, likely as a consequence of low biomass due to the animals’ young age. Thus, all the samples from these time points were excluded from further analysis (Fig. S1). Finally, 244 samples were kept for the rest of analysis.

### Microbial diversity

Rarefaction curves based on rarefied Shannon indices indicated that a sufficient sequencing effort was achieved for all samples, as represented by a plateau in the saturation curves (Fig. S2A,B). However, the observed OTUs metric showed that new ASVs were detected as more reads were analyzed even at a sequencing depth of 11,000 reads per sample, likely indicating transient or a very low abundance of species, as they did not have an effect on the Shannon indices (Fig. S2C,D).

**Figure 2:**
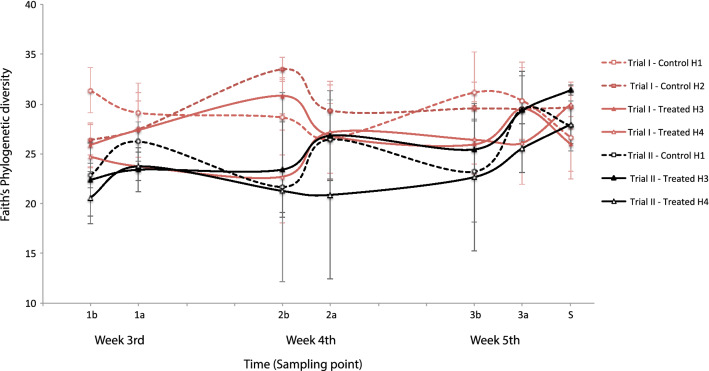
Alpha diversity using Faith phylogenetic diversity metric of samples throughout the experiment per farmhouse and trial. Red lines indicate Trial 1, black lines indicate Trial 2. Dotted lines represent control farmhouses and solid lines are for SalmoFree treated farmhouses. The x-axis indicates the time points for samples taken before (b) and after (a) the delivery of the treatments with their corresponding week of the cycle. The results at slaughterhouse are indicated as (S). Positions on the x-axis are proportional to the time intervals between sampling. Error bars represent the standard deviation of the mean of five cecum samples. This figure was made using Excel for Mac v.16.5 (https://www.microsoft.com/en-ww/microsoft-365/mac/microsoft-365-for-mac).

Alpha diversity analyses for both trials using Faith's phylogenetic diversity and number of observed OTUs suggested a slight increase over time (Fig. S3). This temporal phenomenon is more prominent in Trial 2. However, the variation in diversity between the days of the experiment (17–36) was not high, since Faith indices fall within a comparable range of 20 to 33. This small range might occur if the microbial community is reaching a relatively stable, yet dynamic, state (Fig. 2). This behavior is consistent with a previous report where the microbiome in chickens stabilizes at approximately day 12^35^. Regarding the maturation of the microbiota, the microbial communities have a similar diversity by day 17 (3rd week), at the beginning of the experiment. Between weeks 4 and 5, the microbiome increases its variability and stabilizes again on the last day of the trial, while remaining constant at the slaughterhouse (Fig. 2). Comparing trials, higher alpha-diversity index values (Faith, Shannon and Observed OTUs) were observed in Trial 1 than in Trial 2 (Fig. 2).

Alpha diversity indices changed significantly (Kruskall Wallis test, P < 0.05) in response to the trial; farmhouse; treatment; and, markedly, by the age of the animal. Conversely, the genetic line did not show significant differences (Kruskall Wallis test, P > 0.05) in microbial diversity. Although the treatment showed significant differences, there is no observable pattern that makes it possible to discriminate among treatment groups.

**Figure 3:**
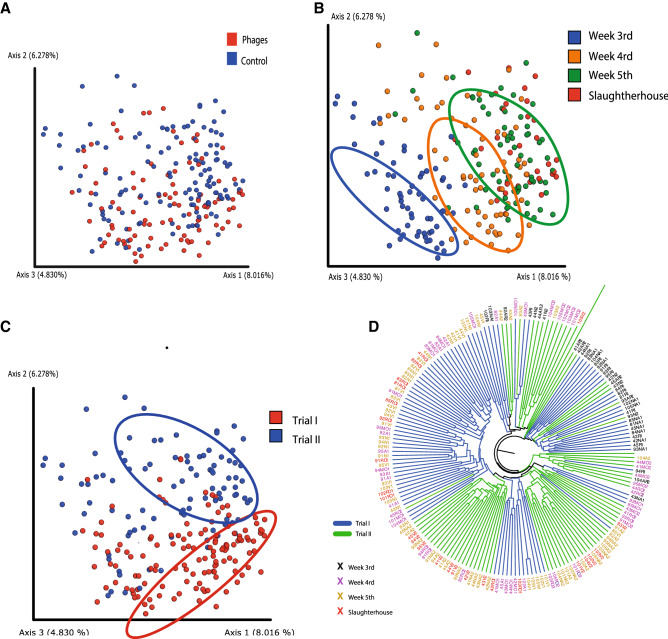
Similarity between the structure of bacterial communities found in cecum of broiler chicken based on Bray Curtis dissimilarity metric. (**A–C**) Principal Coordinate Analysis (PCoA) where samples are colored by treatment (**A**) age of the animals (in weeks) (**B**) and by trial (**C**). Ellipses were manually overlaid to encompass the majority of the sample points of a given feature. (**D**) UPGMA clustering colored by cycle and sampling week. This figure was made using the Qiime2 package version 2018.1156 and its plugins (https://qiime2.org).

Alpha-diversity results were complemented by the beta-diversity comparison of weeks, treatments, and trials (Fig. 3A–D). Principal Coordinates Analysis (PCoA) plots colored by treatment did not reveal any clustering pattern (Fig. 3A) while those colored by age and trial showed that microbial communities were driven mainly by these two variables (Fig. 3B,C). Interestingly, regardless of the trial, the microbial community showed significantly higher similarity at the beginning of the experiment (Fig. 3B,D), and diverged as a function of time, becoming more distant for the second dose of treatments. The trial dependency suggests a high contribution of the environment in the development of the microbiota, which is expected due to the impossibility of controlling all variables in a commercial broiler farm, such as temperature, humidity, feed composition, litter replacement, feeding and antibiotic intervention. The observation of complex but highly similar diversity patterns in older birds coincides with previous studies where microbial communities exhibit similar patterns in chickens once they have reached their marketing age^35^.

### Taxonomic composition of the bacterial community

We performed a general analysis of the taxonomic composition at phylum level of the bacterial community in order to characterize its behavior in a commercial scenario throughout the last stage of the production cycle. We also analyzed the minimum community of microbes that is essential for the host (taxa that were present in more than 50% of samples, will be named “core microbiome”) for both trials, at genus level.

First, the taxonomic composition of trials 1 and 2 indicated that Firmicutes is the most predominant phylum in the gut of broilers (46.4% abundance), followed by Bacteroidetes (37%). Together, both phyla accounted for more than 80% of the relative abundance within the community. A lower proportion of the phylum Epsilonbacterota (4.47%), Proteobacteria (3.2%), and Tenericutes (1.21%) was found, while unassigned bacteria at phylum level accounted for 6.41% of the abundance.

**Figure 4:**
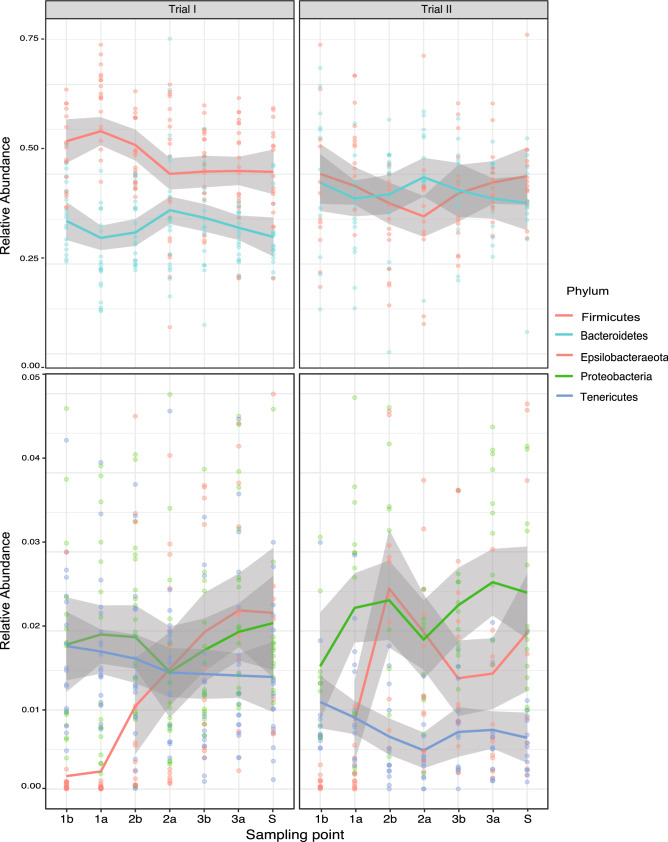
Relative abundance of dominant phyla identified in the microbiome of cecum of broilers. Results are presented discriminated by trial over time, shown as dose days. Sample days are indicated with the nomenclature of the dose number followed by the letter a or b for samples taken before and after the delivery of the treatments, respectively. The results at slaughterhouse are indicated as (S). Upper and lower panels show the range of abundance of Firmicutes and Bacteroidetes compared to other phyla. The color gray indicates the standard deviation of the mean abundance. This figure was generated using Phyloseq package in R v 1.2.5 (https://www.r-project.org/).

Regarding phyla dynamics, Firmicutes and Bacteroidetes remained relatively constant throughout the experiment (Fig. 4), only a slight decrease in Firmicutes, with a corresponding increase in Bacteroidetes was observed the day after the second dose (Day 28 and 27 for Trial 1 and 2, respectively) suggesting a niche complementation between these two phyla. Proteobacteria behaves similarly in the two trials, maintaining relatively constant numbers over time where the lowest point was found after the second dose as well. The presence of phylum Tenericutes bacteria is higher at the beginning of the experiment with a slight reduction over time (Fig. 4). A very low abundance of Epsilonbacteria phylum was found at the beginning of the experiment but it increased rapidly starting in week 4 of the cycle (before the second dose). It then remained constant until the end of the experiment (Fig. 4).

To better understand the shared taxa occurring over time, we analyzed the core microbiome at genus level, discriminated by trial and week (Table 3). When comparing the core microbiome (taxons present in more than 50% of the samples) for both trials over time, it was observed that the genera conforming the broilers’ microbiome are, to a great extent, conserved since most genera (78%) were present in both trials (Table 3). This result is interesting because despite being different in terms of (a) alpha and beta diversity; (b) breeds; and (c) antimicrobial regime, chickens in both trials shared the core genera.

**Table 3:**
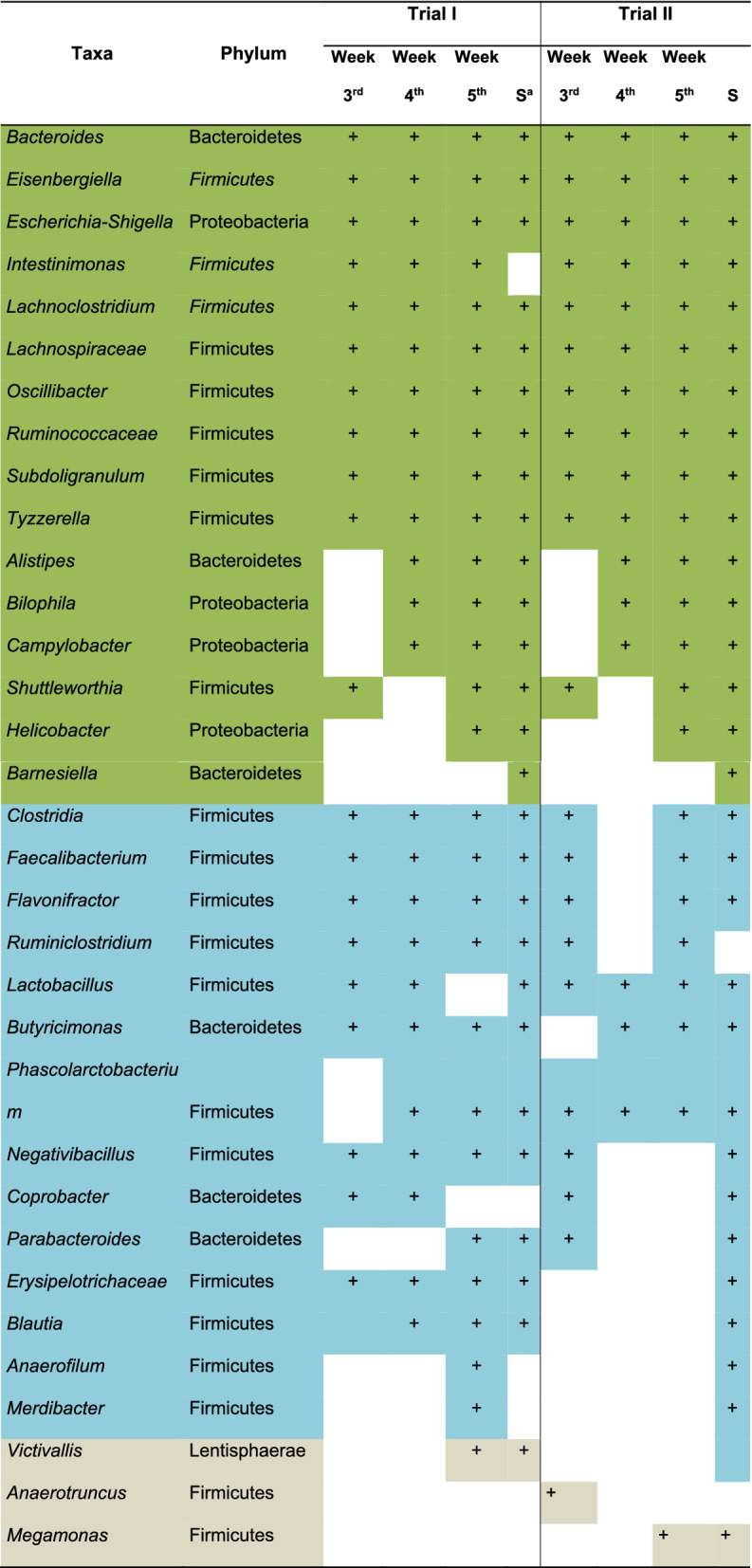
Core microbiome, at genus level, of the last 3 weeks of the broilers’ production cycle and at the slaughterhouse, for the two trials.

Presence (+) of taxa is shown over time with their respective phylum.

A green background indicates taxa that behave similarly in both cycles.

Patterns with variable behavior for both trials are indicated in blue.

Brown corresponds to taxa that are part of the core microbiome (> 50% of the samples) only in the first or second cycle. S shows the results at the slaughterhouse.

Likewise, an analysis of the core microbiome over time showed that the microbiota, during the period examined, was also conserved: 65% of the genera were found in most of the samples during the three weeks (Table 3) and the core microbiome found in broilers at the slaughterhouse contained all the genera found during the experiment. Thus, the most abundant members of the community were maintained as the broilers were transported to the slaughterhouse (Table 3).

### Taxonomic dynamics at the growth stage of the production cycle

**Figure 5:**
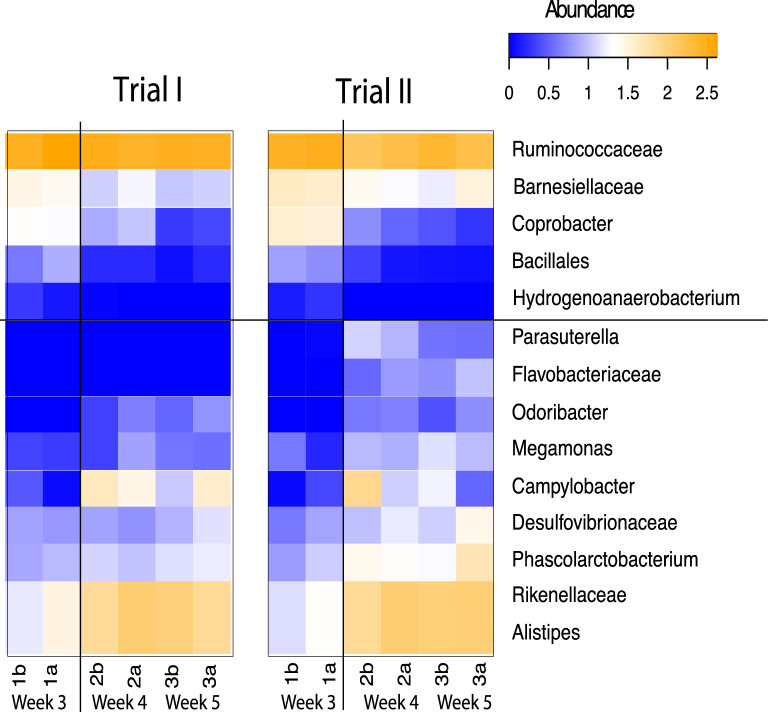
Heatmap built on average abundance values with bacterial taxa found to be significantly associated with the week of the production cycle. Results are discriminated by trial over time of the experiments. Nomenclature of the sample days as shown in Fig. 1. Horizontal black line divides bacteria that decrease after the second week (upper) vs those that increase over time (lower rows). Vertical line marks the end of the 3rd week where most of the significant changes in abundance occur. This figure was made using the Qiime2 package version 2018.1156 and its plugins (https://qiime2.org).

The differential analysis discriminated by week allowed the identification of some key species in the adaptation of the microbiota during the broilers’ growth phase. For instance, in both trials, several taxa revealed an increase, over time, in the abundance of *Alistipes*, *Rikenellaceae*, *Phascolarctobacterium*, *Desulfovibrionaceae and Megamonas*, while *Bacillales*, *Coprobacter*, *Barnesiellaceae* and *Ruminococcaceae* presented a decrease in abundance. Another genus with an intriguing abundance was *Odoribacter,* which increased between week 3 and 4 and then remained relatively constant until the end of the cycle. In contrast, *Hydrogenoanaerobacterium* is only detected at the beginning of the cycle (week 3) and then disappeared (Fig. 5).

### Variation in abundance of the microbial taxa

**Figure 6:**
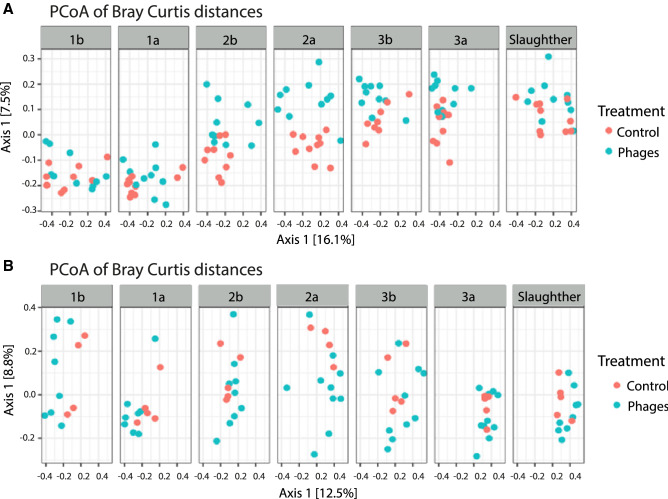
Principal Coordinate Analysis (PCoA) representing the similarity of bacterial communities found in the cecum of broiler chicken treated with (phages) and without (controls) the bacteriophage cocktail, measured using the Bray Curtis metric. Axes represent the coordinates of the samples in Axis 1 and 2 of the corresponding PCoA plots. Blocks of samples are divided by dose day and nomenclature as described in Fig. 1. Upper panel display Trial 1 (**A**) and the lower panel Trial 2 (**B**). This figure was generated using Phyloseq package in R v 1.2.5 (https://www.r-project.org/).

As some of the most important factors affecting the structure of the microbiota was the trial (Fig. 2), a differential analysis was carried out separately for each trial in order to define the differences between the farmhouses in particular those receiving the phage cocktail treatment and those that didn’t. This analysis was an independent PCoA analysis by Trial, whose results revealed a separation by treatment after the second dose. However, this trend disappeared towards the end of the experiment. This separation was clearer for Trial 1 (Fig. 6), although a more compact clustering of all samples can be observed towards the final days of the experiment for both trials.

**Figure 7:**
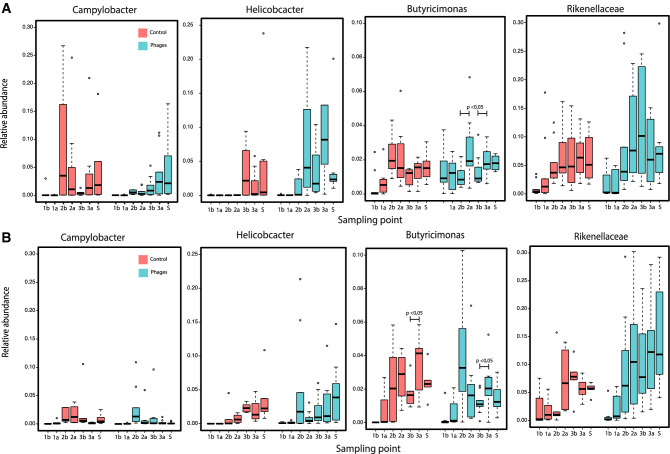
Relative abundance of Bacterial taxa significantly associated with the treatment. Results are discriminated by trial over time, shown as sampling point as in Fig. 1. Results are presented by Trial 1 (**A**) and Trial 2 (**B**). Control groups are depicted in salmon while treated groups are shown in blue. The body of the box plot represents the first and third quartiles of the distribution and the median line of abundance of all ASV assigned to the corresponding genera. The whiskers extend from the quartiles to the maximum or minimum data within 1.5× interquartile range, with outlayers beyond. ANOVA test P < 0.05 shows significant differences in microbial abundance of Butyricimonas before and after the dose of phages. This figure was generated using Phyloseq package in R v 1.2.5 (https://www.r-project.org/).

The differential abundance analysis for the different doses revealed four genera as being significantly associated with the treatment: the taxa *Campylobacter*, *Helicobacter*, *Rikenellaceae* and *Butyricimonas* (Fig. 7). *Campylobacter* in the first trial appeared between the first and second dose in both control and treated samples; however, the abundance in treated farmhouses increased slowly, in contrast to the abrupt increase in the control groups during the second dose with a subsequent decrease, leading to a final convergence (for treatments and controls) in abundance at the end of the experiment. Interestingly, in contrast to the under-representation of *Campylobacter* in treated samples, the closely related *Helicobacter* seemed to increase in abundance. *Butyricimonas* increased significantly with respect to the control after the second and third doses in Trial 1 (ANOVA, P < 0.005). Similarly, the abundance of *Rikenellaceae* increased after the second doses and it is significantly different, compared to the control group (Fig. 7A).

Abundance patterns of particular taxa displayed similarities in both trials. In both cases, the abundance of *Rikenellaceae* was greater in treated chickens than it was in the control in the last week of the experiment. Even though the tendency of an increased abundance over time is observed for treatment groups in both trials, Trial 2 presented overall lower abundances than Trial 1, as seen for *Campylobacter* and *Helicobacter* (Fig. 7B). In addition to these taxa, the *Parasutterella* genus that was not detected in Trial 1, increased in abundance over time in Trial 2.

Given the differential abundance of *Helicobacter* seen for the treatment groups and since some species of the *Helicobacter* genus are considered human and animal pathogens, a further characterization of the ASVs assigned to *Helicobacter* was performed. These ASVs were aligned with the collection of *Helicobacter 16S rRNA* gene sequences deposited in the NCBI Refseq collection. The bioinformatic analysis identified these ASVs as *Helicobacter pullorum* (Fig. S4).

### Enterobacteriaceae abundance analysis

Given that the traditional methods for taxonomic assignment lack the resolution to discriminate among Enterobacteriaceae for the sequenced segment of the *16S rRNA* gene, we implemented a more detailed analysis allowing the identification of one ASV as *Salmonella* from the 32 assigned to Enterobacteriaceae. This ASV was clustered in the clade that grouped *Salmonella enterica* subsp. *enterica* and separated from the clades that grouped *Salmonella bongori* and *Escherichia coli*-*Shigella* (Fig. S5). Thus, we established that this ASV corresponded to *Salmonella* sp. The other ASVs assigned to the Enterobacteriaceae family were grouped in the *E. coli-Shigella* clade.

The abundance of the *Salmonella*-related ASV across samples was rather low, in a range of 2.5 × 10^–5^ to 6.8 × 10^–4^, and close to our estimated confidence detection limit for the method (1 × 10^–4^). The ASV was also detected in a small number of samples (n = 22). Thus, further inferences based on its abundance were discarded. In addition, no correlation was found between the abundance of this ASV and the molecular detection of *Salmonella* in the samples (P > 0.01) (Fig. S6). The information regarding the molecular detection of *Salmonella* was obtained from the previously published results^32^. This result indicated that, despite identifying specific regions within the sequenced gene fragment to discriminate *Salmonella* from the other members of the Enterobacteriaceae family, due to its very low abundance, a deeper sequencing would be necessary to quantify it within accurate detection levels.

## Discussion

The purpose of this study was to characterize the cecal microbiota of broiler chickens in a commercial setting during a phage therapy treatment, using the *Salmonella* phage cocktail SalmoFree. Previously published results showed the efficacy of the phage cocktail in reducing Salmonella under these conditions^32^. However, it also showed a basal level of cross-contamination of the phage cocktail in the control samples, hindering any conclusions regarding the effect of phages in the microbial community structure. Regardless, our results suggest a process of normal microbiota maturation characterized by a transition towards a higher diverse community. This observation was independent of the farmhouse and treatment applied.

Our results also suggest that the behavior of the microbiota between 17 and 36 days of age, when chickens are in the grower phase, is similar to what has been previously reported in experimental chickens reared in controlled environments^35,36^. For instance, our observations support the stabilization dynamic of the microbiota at this developmental stage. It was also confirmed that the microbial community in older chickens is similar to the one observed at slaughter age (Figs. 2, 3). Also in agreement with previous studies, the age of the animal was the variable that had the greatest influence on the variation in the microbiota^35,37^. This provides key evidence suggesting that microbiota approximations conducted under controlled environments do not differ largely from farming conditions. This result has a great impact, as it validates the use of controlled environments as proxies to what could be happening under farming conditions since the development of large-scale assays is often more expensive and challenging.

Analyses of similarities among communities made it possible to identify two main moments of microbiota development in the grower cycle (Fig. 3). First, the 3rd week, which was the first week of the experiment and coming right after the change made to the grower diet, is where the community seems to be more uniform and with a significantly higher abundance of bacteria such as *Ruminococcaceae*, *Bacillales, Coprobacter, Hydrogenoanaerobacterium* and *Barnesiellaceae* (Fig. 5) compared to *Parasuterella* and *Flavobacteriaceae*. Second, in the 4th and 5th week, there is a higher variation in abundance and diversity, which could be attributed to the change in diet. During those weeks, the microbiota becomes populated by *Phascolarctobacterium*, *Desulfovibrionaceae*, *Megamonas*, *Odoribacter*, *Rikenellaceae* and *Alistipes*. These bacteria could represent biomarkers of microbiota maturation under rearing conditions (altitude: 1230 m.a.s.l.; litter composition: ground; average of no. of chickens/m^2^: 13.86; average of area house in m^2^: 645.61). Nevertheless, further studies are necessary to confirm the generalization of our current results to other farms and conditions.

Analysis of the core microbiome identified members that were reported previously in the literature as being part of the most abundant genera in the microbiome of chicken cecum^35^. In one previous study, authors performed a comprehensive day-to-day microbiome analysis of the chicken cecum from day 3 to 35 using experimental chickens in a controlled environment. They identified the most abundant genera to be *Escherichia*, *Shigella*, *Eisenbergiella*, *Ruminiclostridium*, *Flavonifractor*, *Anaerotruncus, Faecalibacterium*, *Lachnoclostridium*, *Megamonas*, *Intestinimonas*, *Shuttleworthia, Subdoligranulum*, *Tyzzerella*, *Lactobacillus*, *Blautia* and *Erysipelotrichaceae*. We worked in a productive set up and found these genera in our core analysis (Table 3).

These commonly abundant members of the microbiota identified key players in the last stage of the production cycle (Fig. 3) (Table 3). These microorganisms may be responsible for important metabolic processes in the intestines of broiler chickens. Among these, there are some important degraders of complex polysaccharides and producers of short chain fatty acids (SCFA). For instance, *Eisenbergiella*, *Lachnoclostridium* of the *Lachnospiraceae* family play an important role in the production of butyrate which is the preferred energy source for the gut epithelial cells^38^. Another butyrate producer found in the core was *Intestinimonas*^39^. *Megamonas* and *Bacteroides* were detected as well; these bacteria are known to produce propionate as the main end product of the degradation of complex plant polysaccharides. Although propionate is a less preferred energy source than butyrate, its production might represent an efficient balance between energy acquisition from available nutrients and sustained growth^37^. Other bacteria present in the core microbiome involved in producing SCFA were *Subdoligranulum*, *Faecalibacterium, Alistipes*, *Coprobacter*, *Blautia* and *Butyricimonas*^40^.

On the other hand, *Campylobacter*, *Helicobacter* and *Megamonas* are bacteria carrying hydrogenases that can serve as hydrogen sinks that facilitate succinate production^41^. Succinate is an important metabolite in both host and microbial processes^42^. Meanwhile, the presence of *Oscillibacter*, a Clostridium cluster IV member, has been identified as an anaerobe producer of valerate and associated with diet-induced obesity^43^. Surprisingly, *Bifidobacterium*, a butyrate producer, was not detected while it has been reported consistently as a dominant member of the chicken microbiota^35–37^.

Comparison analyses between treated and control farmhouses must be conducted with caution, because of the conditions and characteristics of the current trials (variation of temperature, humidity, feed composition; the antibiotic intervention; and phage cross-contamination). However, some significant differences were observed. Particularly interesting is the evidence showing the reduction of *Campylobacter* in treated farmhouses. *Campylobacter* is considered an important food-borne pathogen associated with the consumption of poultry products and is of great importance in terms of public health^44^. However, when this opportunistic pathogen is highly abundant in chickens, it has been demonstrated to cause damage to the gut^45^. Furthermore, the correlated increase in *Helicobacter* abundance with the decrease in *Campylobacter* supports the proposal of a competitive dynamic between these two genera^46^. It is also important to mention that we showed that the *Helicobacter* present correspond to *Helicobacter pullorum* and not *Helicobacter pylori,* the latter of which could constitute an important human health risk. The increase of *Butyricimonas* and *Rikenellaceae* following treatment is noteworthy. These two genera are reported as beneficial bacteria in chickens due to their enrichment in samples treated with probiotics^47^. The cause of this variation between farmhouses, and whether it is driven as an indirect effect of the phage treatment will required further validation.

## Conclusion

This study characterized the development of the cecal microbial community in broiler chickens at a production scale. It showed a normal microbiota maturation process evolving to a higher diversity in the ceca of broilers. It also showed a stabilization of this microbiota at the end of the production cycle. Our analyses revealed that the core microbiome in broiler chickens contain key species such as *Eisenbergiella* and *Lachnoclostridium* that are important for the microbiota adaptation in the last stage of the production cycle. Our study further showed that the use of the SalmoFree cocktail didn’t had a significant effect in the maturation of the microbiome. Together, this study shows the feasibility of following the cecal microbiome under farming conditions in broiler chickens. However, due to the different variables affecting animals at a production scale, it remains challenging to test specific effects of further disturbances and treatments such as a phage cocktail.

## Methods

### Experimental design

This study was approved by the Institutional Committee on the Care and Use of Experimental Animals (CICUAL) at Universidad de los Andes, Ref. CICUAL 15-008, in the framework of Colombian Law 84/89 and Resolution 8430/93. We confirm that all experiments were performed in accordance with relevant guidelines and regulations, as well as in compliance with the ARRIVE guidelines (https://arriveguidelines.org).

Two field trials under commercial rearing conditions were carried out in a commercial broiler farm in Colombia^32^. The farm belongs to an integrated poultry company that typically handles the entire poultry production and processing cycle (hatching, feed, production, processing, and marketing).

Four production houses (labeled as houses 1, 2, 3 and 4) were selected based on the existing record of *Salmonella* presence detected during two previous production cycles (data not shown). Chickens in houses 1 and 2 were treated with a control suspension (see below) whereas houses 3 and 4 were treated with the SalmoFree bacteriophage cocktail. Chickens from the treated houses were separated from the controls by a distance of 300 m approximately. The farmhouses contained an average of 13.86 chickens/m^2^ with an average area per house of 645.61 m^2^. All animals were co-housed on the same day and at the same age. Animals were provided with water and feed ad libitum. Additional information about the size of the houses, breed line, sex, number of chickens, biosecurity protocols and antimicrobial therapy were previously published in Clavijo et al.^32^. Houses 1, 2, 3 and 4 correspond to 4, 8, 9 and 10 in Clavijo et al., respectively.

The broiler production cycle in Colombia is carried out in two stages. The first stage comprises the period from which 1-day old chickens are received at the farm (day 1), until day 13–17. Chickens at this stage are fed with a starter diet. Following this diet, the grower stage in which the chickens receive a grower diet, spans from days 14–18 until day 35–42. Variations in the length of each period depend on several factors (market demand, weight of the chickens, schedule at slaughterhouse, among others). At the end of the second stage, chickens are sent to the slaughterhouse.

SalmoFree and the control suspensions were delivered to the animals in their drinking water during the grower stage in three doses (one a week): at the beginning (day 18 for both trials), in the middle (day 27 and 26 for the first and second trial, respectively), and one day before slaughter (day 35 and 34 for the first and second trial, respectively) (Fig. 1). Between days 15 and 21 of the first trial, treated houses (3 and 4) received an emergency antibiotic intervention (not planned within the experimental design). This intervention was performed by the veterinarian in charge due to the high rate of chicken mortality observed in house 4. Despite the fact that only house 4 showed high mortality, antibiotic was applied to houses 3 and 4 because the chickens came from the same egg batch (Fig. 1).

### Phage and control treatment preparation

The methodology used is described in detail in Ref.^32^. Briefly, SalmoFree was prepared following a standard liquid lysate procedure using *Salmonella* Enteritidis s25pp^48^. The strain was donated by Dr. Pilar Donado from the Colombian Integrated Program for the Antimicrobial Resistance Surveillance (COIPARS–CORPOICA). *Salmonella* Enteritidis s25pp was grown on nutritive agar (Sharlau) media for 18 h at 37 °C. The phage cocktail was prepared individually for each of the six phages in nutritive broth (Sharlau), using an MOI of 0.1 following the standard liquid lysate procedure^48^. Each lysate was centrifuged at 4 °C at 13,000×*g* for 20 min and the supernatant was filtered through a 0.22-µm filter. Individual phages were mixed to obtain a suspension of 10^10^ PFU/mL immediately before administering each treatment. Approximately 5.5L of each phage was produced per trial and stored at 4 °C. Quality control of the cocktail and concentration was verified as previously reported^32^.

The suspension for the control treatment was prepared using a fully-grown *Salmonella* culture that was lysed by adding chloroform at a final concentration of 0.1% (v/v). The lysate was centrifuged at 4 °C at 13,000×*g* for 20 min and filtered through a 0.22-µm filter. The final suspension was verified to be free of bacteria, by plating 100 µL of the suspension on nutritive agar (Sharlau) and incubating it at 37 °C for 24 h. The absence of any type of growth was verified. This control allowed the estimation of whether the cell residuals found in a normal lysate have any effect on the observed results.

### Delivery of the treatments to the animals

The drinking water supply was removed 30 min before administering the treatments. This is a regular practice in the poultry production applied in order to facilitate the uptake of any treatment due to the temporary shortage of hydration; it does not endanger the health of the chickens in any way. The water supply tanks from each farmhouse can store up to 1000 L of water. SalmoFree and control suspensions were added to these tanks in a 100:1 water to treatment ratio. Thus, the final concentration of the phage suspension was 10^8^ PFU/mL. Treatments were delivered to the animals for 2.5 h, which is the time needed for the animals to drink the full 1000 L water supply. Due to the total number of animals in each house, it is impossible to administer the treatment individually or to guarantee that every animal received the same dose. However, adding treatments in the drinking water after a short fasting period is a common practice used in commercial farms for the application of other products such as vaccines, and probiotics among others.

### Sampling methods

Cecum samples were taken 1 day before and one day after delivery of SalmoFree treatments, as shown in Fig. 1. Samples were also collected 1 day before the start of the growing phase (day 15 and 14 for the first and second trial, respectively) and at the abattoir, after the slaughter. For all sampling points, five female chickens from each of the four houses were randomly selected. These chickens were sacrificed and their ceca were removed in the most aseptic way possible on the farm. Each cecum was placed into a sterile plastic bag (Nasco, USA) and transported in liquid nitrogen to the laboratory where they were stored at − 80 °C until processed for DNA extraction. A total of 320 samples were collected corresponding to 160 per trial (5 ceca per house, per 4 houses, per 8 sampling days). Since sampling days occurred at different points of the growth cycle for Trial 1 and Trial 2 (Fig. 1), we renamed the samples according to each treatment dose, for ease of comparison. The sampling point corresponds to the number of doses (1–3) followed by a letter indicating whether it was taken one day before (b) or after (a) the corresponding dose.

In addition to the ceca samples, individual cloacal swaps samples were taken in order to detect *Salmonella* using a genus-specific PCR as described in Clavijo et al.^32^. Each individual swab corresponded to the same individual chicken sacrificed for cecum extraction.

### DNA extraction and 16S rRNA gene amplification

From ceca samples, 180–200 mg of the cecal content were aseptically collected in dry ice, making sure that the samples did not thaw. The samples were processed immediately using the QIAamp DNA Stool Mini Kit (Qiagen, Valencia, CA) according to the manufacturer’s instructions. Total DNAs were measured using a Nanodrop ND-1000 spectrophotometer (Thermo Scientific, Wilmington, USA) to assess the DNA quality and were quantified using a Qubit fluorometer (Life Technologies, Paisley, UK). After DNA quantification, samples were diluted with elution buffer (Qiagen, Hilden, GM) to a concentration of 5 ng/µL. The V4 hypervariable region of the *16S rRNA* gene was amplified using the following primers:

- Primer1 5′ TACACGACGCTCTTCCGATCT**GTGCCAGCMGCCGCGGTAA 3**′ and;
- Primer2 5′ AGACGTGTGCTCTTCCGATCTG**GACTACHVGGGTWTCTAAT 3**′.

The bold region of the primers corresponds to the universal 515F and 806R primers. Each PCR reaction was set for 20 µL final volume and contained: buffer (1×), dNTPs (10 mM), Primer1 (10 µM), Primer2 (10 µM), Phusion High-Fidelity DNA Polymerase (0.02 U/µL) and 1.5 µL DNA (7.5 ng on average). The temperature profile for the reaction was as follows: Initial denaturation 94 °C for 3 min; 30 cycles at 94 °C for 45 s, 56 °C for 30 s and 72 °C for 30 s; and the final extension at 72 °C for 7 min. The PCR procedure was carried out in triplicate with a negative control in which water was added instead of the DNA sample. PCR products were visualized using Gelred through 1.5% (w/v) agarose gel electrophoreses. Finally, once amplification was confirmed, the PCR triplicates were mixed in one pool and kept at − 20 °C until further processing.

### Libraries preparation and sequencing

Following the first amplification, a second PCR was carried out using a pair of primers containing the Illumina adapters and indexes, for bioinformatic demultiplexing of the samples. The sequences of the primers are as follows:

- Primer3: 5′ AATGATACGGCGACCACCGAGATCTACACNNNNNNNNNACACTCTTTCCCTACACGA
- Primer4: 5′ CAAGCAGAAGACGGCATACGAGATNNNNNNNNGTGACTGGAGTTCAGACGTGTG

The underlined region corresponds to the location of a particular index (the sequences for all primers are presented in Table S1). Each sample was amplified with a pair of primers with a different sequence in this region in order to pool all the samples in the same sequencing run and demultiplexing them afterwards. The second PCR was performed by adding 5 µL of the pooled product from the triplicate per sample of the first PCR to a mixture with 4 µL of water, 10 µL of GoTaq Green Master mix (Promega) and 0.5 µL of each Illumina index primer (0.25 µM) which was amplified using the following PCR conditions: 3 min at 94 °C, and 12 cycles of 45 s at 94 °C, 60 s at 55 °C and 30 s at 72 °C and a final period of 7 min at 72 °C and kept at 4 °C. PCR products were purified using 18 µl of AMPure beads (Beckman Coulter) and eluting samples with 15 μL of Tris buffer (10 mM, pH 8.5). The concentration of purified amplicons was determined with the Qubit fluorometer (Life Technologies, Paisley, UK) followed by pooling all the libraries into equimolar concentrations. Paired-end sequencing (2 × 250) of this pool was conducted on an Illumina MiSeq platform at Washington University in Saint Louis, Center for Genome Sciences and Systems Biology.

### Bioinformatic and statistical analyses

Sequences were pre-processed, quality filtered and analyzed using QIIME2 version 2018.11^49^ (https://qiime2.org) and its plugins. The input files used were the demultiplexed paired-end fastq files generated in Casava format (Illumina) and a mapping file. Raw sequencing data was imported and demultiplexed through the Casava 1.8 paired-end demultiplexed fastq protocol. Following this, adapters were removed using the cutadapt plugin^50^ and subsequently the fastq sequences were merged using FLASH software^51^. DEBLUR software package^52^, included in QIIME2, was used for modelling and correcting Illumina sequences. This process integrates chimera removal, truncation of reads and the collapse of reads into Amplicon Sequence Variants (ASVs). All parameters were used by defaults except for read truncation: -p-trunc 214. The method’s detection limit (1 × 10^–4^) was established based the maximum number of rarefied reads per sample (10,000 per sample) implying that any ASV at an abundance of less than 1/10,000 will not be detected.

ASVs were filtered using QIIME2 q2-feature-table filter features command, keeping only features with a frequency higher than 10, in general corresponding to a minimum relative abundance of 0.001 and present in at least 2 samples. A second filtration was conducted retaining samples with more than 3000 sequences after ASV filtration. Taxonomy assignment to the ASVs was performed using QIIME2 q2-feature-classifier plugin and the Naïve Bayes classifier that was trained on the SILVA database (version SSUParc_100)^53^.

Alpha- and beta-diversity analyses were performed with the q2-diversity plugin at a sampling depth of 10,000. Alpha diversity was calculated using Shannon’s diversity index, observed OTUs and Faith’s Phylogenetic diversity. Kruskall–Wallis test was used to test for differences in mean alpha-diversity between experimental treatments and trial, farmhouse, genetic line, sampling point, and dose variables. Distance matrices for beta-diversity were constructed using Bray Curtis and weighted Unifrac metrics. Permutational multivariate analysis of variance (PERMANOVA, P < 0.05) using group significance command was used to analyze spatial variation in beta-diversity and the effects of experimental treatments and the other variables. Unweighted Pair Group Method with Arithmetic Mean (UPGMA) clustering analysis based on Bray Curtis and weighted UniFrac distance was also used. Significant enrichment of taxa between the groups was assessed with the ANCOM test in QIIME2. Specific analyses were carried out by week, collapsing samples between cycle days 15–21 in week 3, days 22–28 in week 4, and days 29–36 in week 5 (Fig. 1). The phyloseq package^54^ in R was used to determine the core microbiome and to analyze the abundance of the predominant phyla over time.

### Bioinformatic Salmonella analyses

Current taxonomical assignments are based on k-mer frequency or percent similarity to reference sequences for assignment; however, for closely related sequences, a conservative approach and assignment at a higher taxonomical level were employed for small variations. Given that none of the identified ASVs was assigned to the *Salmonella* genus, but several of them were annotated as Enterobacteriaceae family, the next step was to determine whether some of those ASVs in the feature file were closely related to *Salmonella*. For this purpose, a collection of reference *Salmonella*, *Escherichia coli* and *Shigella 16S rRNA* genes deposited in the NCBI Refseq collection were retrieved. These sequences (Table S2) were used along with the sequences of ASVs assigned to the Enterobacteriaceae family for multiple alignment using MUSCLE^55^. Next, the alignments were manually inspected and edited using the sequence editor Jalview (version 2)^56^ to identify specific positions that discriminate the *Salmonella* group. A maximum-likelihood phylogenetic tree was then constructed using FastTree version 2.1^57^ and visualized in Figtree^58^. Based on this analysis, it was possible to identify the ASV that most likely corresponds to *Salmonella*.

A similar characterization of the ASVs assigned to *Helicobacter* was performed since some species of the *Helicobacter* genera are considered human or animal pathogens. These ASVs were aligned with the collection of *Helicobacter 16S rRNA* gene sequences deposited in the NCBI Refseq collection (Table S3) and the same procedure as above was performed.

## Supplementary Information

Supplementary Figures.

Supplementary Tables.

Supplementary Information.

## Data Availability

Sequence files and metadata for all samples used in this study have been deposited at the European Nucleotide Archive (ENA) (https://www.ebi.ac.uk/ena/) under the study Accession No. PRJEB32104. A record of all statistical analysis is included as Additional File 3.

## Abbreviations

ASV: Amplicon sequence variants
PCR: Polymerase chain reaction
