# Supplementary material for: The gut microbiota of chickens in a commercial farm treated with a Salmonella phage cocktail
Source: Sci Rep. 2022 Jan 19;12:991. doi: 10.1038/s41598-021-04679-6 (PMC8770602; doi:10.1038/s41598-021-04679-6)

Supplementary material

Salmonella Phage cocktail, its effects and benefits on the gut of chickens in a commercial farm.

Viviana Clavijo[a,]; Tatiana Morales[b]; Martha Josefina Vives Flores[b] and Alejandro Reyes Muñoz[a,c,d*]

a. Grupo de investigación en Biología Computacional y Ecología Microbiana, Universidad de los Andes, Bogotá, Colombia. Cra 1 #18A-12, Bogotá, Colombia
b. Centro de Investigaciones Microbiológicas, Universidad de los Andes. Carrera 1 Este #19A-40, Bogotá, Colombia
c. Max Planck Tandem Group in Computational Biology, Universidad de los Andes, Carrera 1 Este #19A-40, Bogotá, Colombia
d. Center for Genome Sciences and Systems Biology, Washington University School of Medicine, Saint Louis, MO, 63108, U.S.A

* Corresponding author: a.reyes@uniandes.edu.co, Telephone: +57 1 3394949 Ext. 2763

Supplementary figures

Figure S1. Principal Coordinate Analysis (PCoA) of the raw data representing the similarity of bacterial communities found in cecum of broilers. PCoA shows results by dose day and by cycle. Trial 1 (red), Farmhouse 2 in Trial 2 (blue), other farmhouses in Trial 2 (green). The nomenclature of the sampling point corresponds to the dose followed by the letter b (before) or a (after). Day 0 represents samples collected on the first day of the experiment at age 14-15 of the chickens. This figure was generated using Phyloseq package in R v 1.2.5 (https://www.r-project.org/)

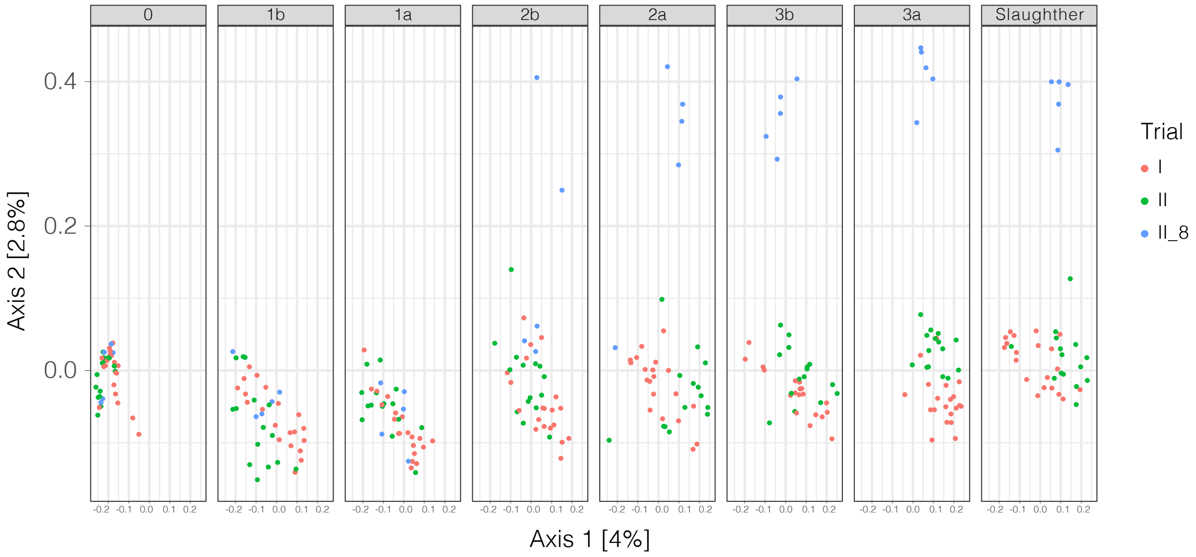

Figure S2. Alpha diversity using Shannon indexes (A, B, C) and Observed species (D, E, F) discriminated by sample (A, D); Trial (B, E) and sampling point (C, F). This figure was made using Prism 9 (https://www.graphpad.com/)

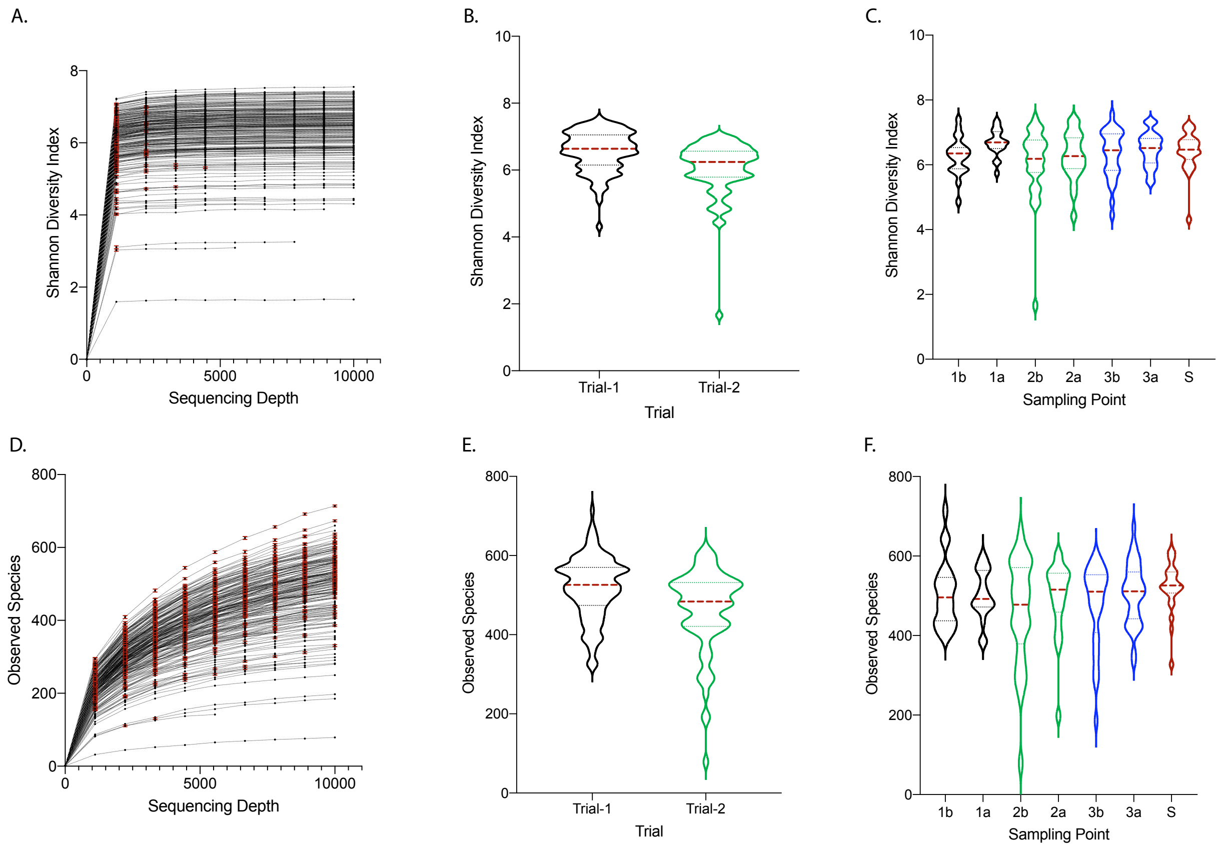

Figure S3. Alpha diversity as measured by A) Faiths phylogenetic distance and B) observed OTUs throughout the experiment. Note the consistent increase of the average over time. This figure was made using the Qiime2 package version 2018.1156 and its plugins (https://qiime2.org)

A)

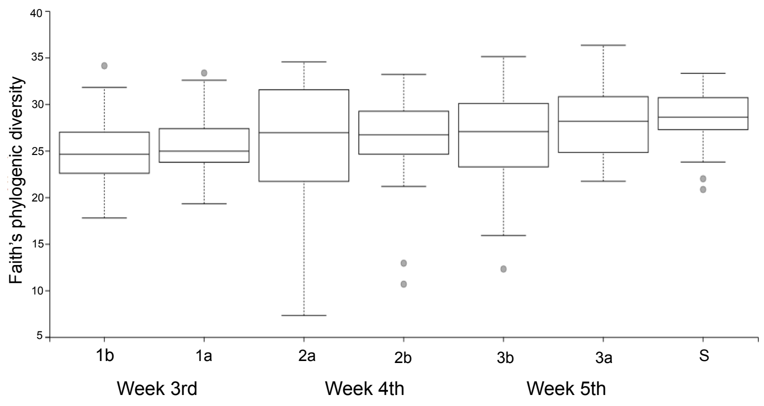

B)

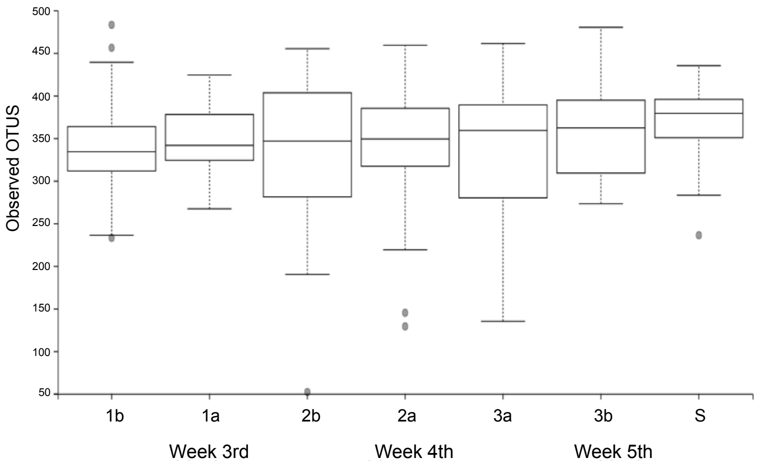

Figure S4. Dendrogram representing the similarity between different species of *Helicobacter* (black) and ASVs assigned as *Helicobacter* (red). Notice that *Helicobacter pullorum* sequences are highlighted in green while *Helicobacter pylori* are in blue. In bold are 4 sequences from F. rappini used as an outgroup. This figure was generated using FigTree v 1.4.4 (http://tree.bio.ed.ac.uk/software/figtree/)

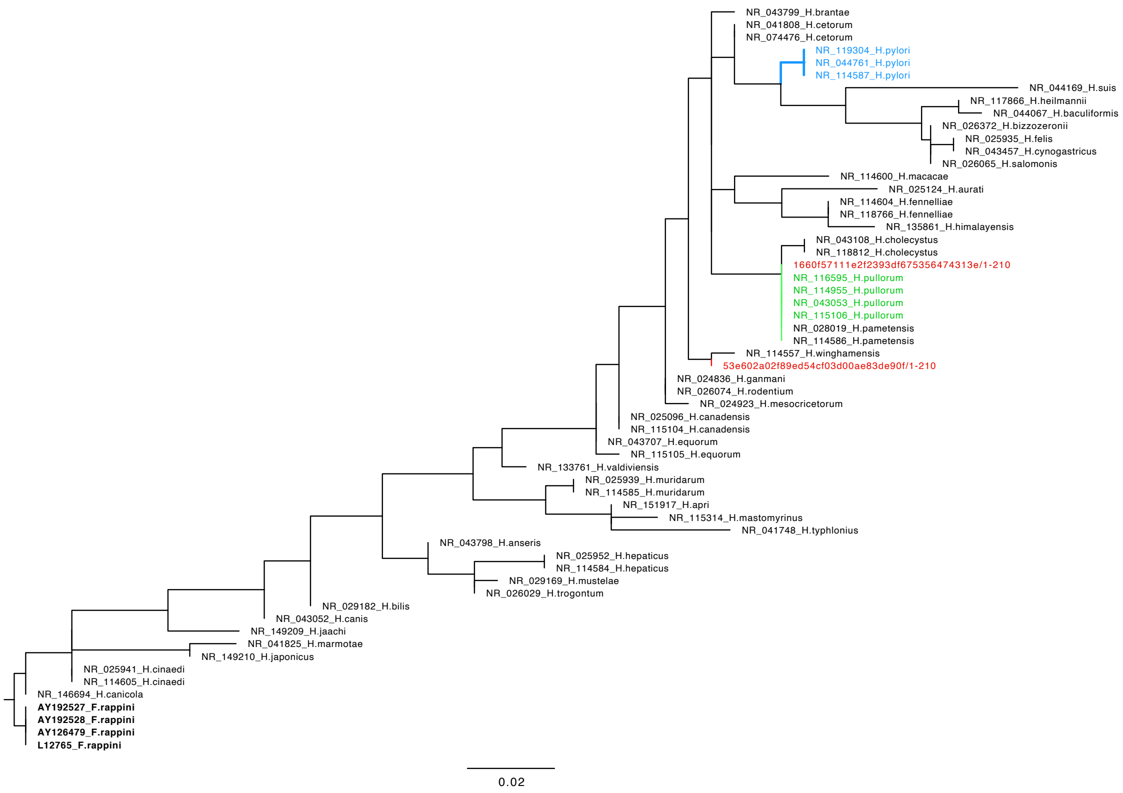

Figure S5. Dendrogram representing the similarity between *Escherichia coli* (green), *Shigella* sp. (purple), *Salmonella enterica* (blue) and *Salmonella bongori* (red) and ASVs assigned as Enterobacteriaceae (black). This figure was generated using FigTree v 1.4.4 (http://tree.bio.ed.ac.uk/software/figtree/)

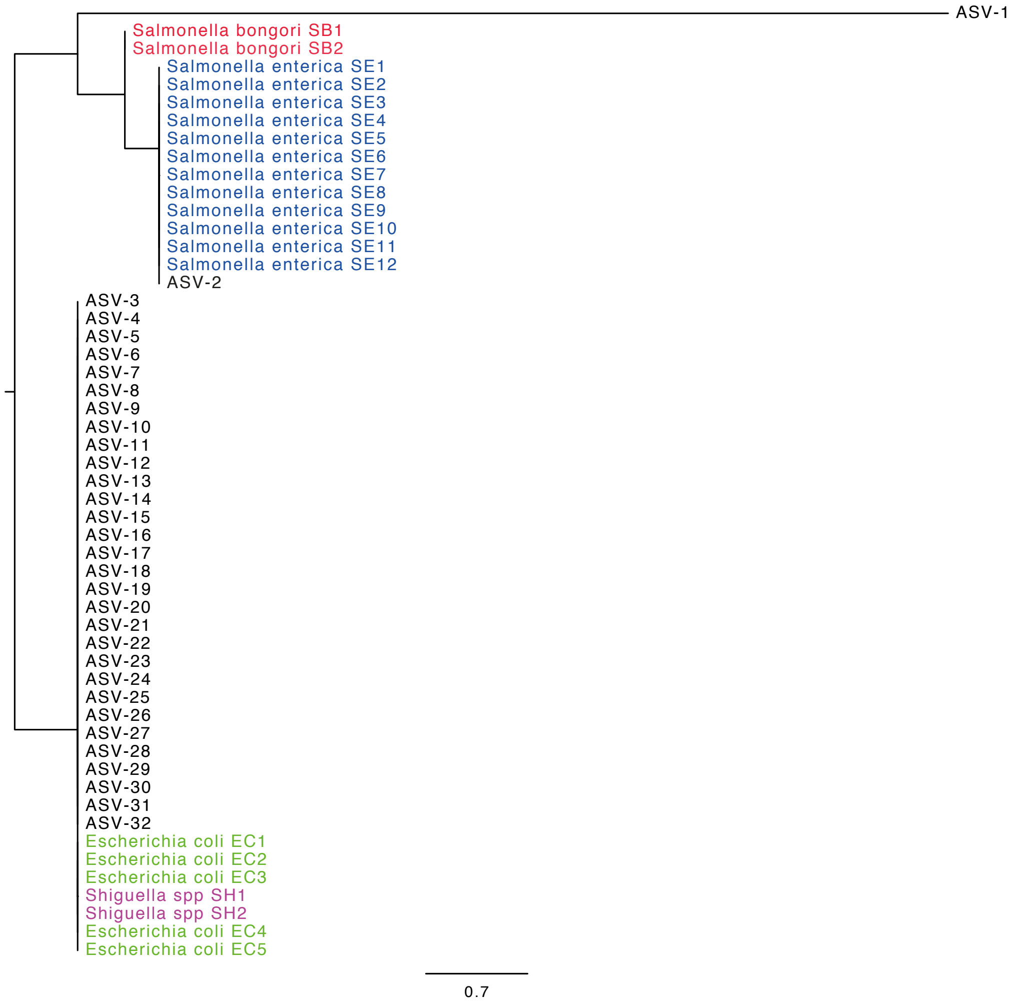

Figure S6. Boxplots showing the abundance of the ASV corresponding to *Salmonella* sp. within the different treatments (two-control and two phage-treated farmhouses). Note that the black line corresponds to the average showing that most of the data has zero abundance. The number of samples per farmhouse is indicated below. This figure was generated using Phyloseq package in R v 1.2.5 (https://www.r-project.org/)

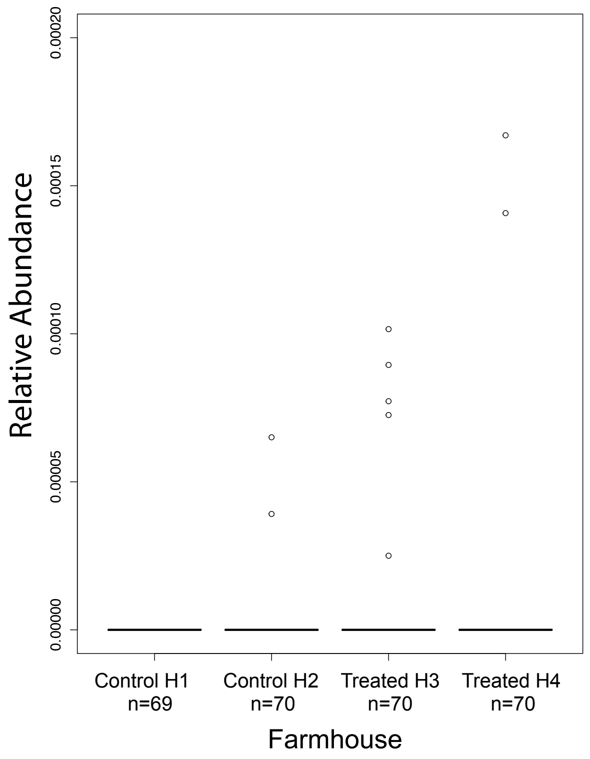

Supplement: Supplementary file 1 — Supplementary Figures. [file 41598_2021_4679_MOESM1_ESM.pdf]
