# Supplementary material for: The gut microbiota of chickens in a commercial farm treated with a Salmonella phage cocktail
Source: Sci Rep. 2022 Jan 19;12:991. doi: 10.1038/s41598-021-04679-6 (PMC8770602; doi:10.1038/s41598-021-04679-6)
Supplement: Supplementary file 2 — Supplementary Tables. [file 41598_2021_4679_MOESM2_ESM.pdf]

Supplementary material

Salmonella Phage cocktail, its effects and benefits on the gut of chickens in a commercial farm.

Viviana Clavijo[a]; Tatiana Morales[b]; Martha Josefina Vives Flores[b] and Alejandro Reyes Muñoz[a,c,d*]

a. Grupo de investigación en Biología Computacional y Ecología Microbiana, Universidad de los Andes, Bogotá, Colombia. Cra 1 #18A-12, Bogotá, Colombia
b. Centro de Investigaciones Microbiológicas, Universidad de los Andes. Carrera 1 Este #19A-40, Bogotá, Colombia
c. Max Planck Tandem Group in Computational Biology, Universidad de los Andes, Carrera 1 Este #19A-40, Bogotá, Colombia
d. Center for Genome Sciences and Systems Biology, Washington University School of Medicine, Saint Louis, MO, 63108, U.S.A

* Corresponding author: a.reyes@uniandes.edu.co, Telephone: +57 1 3394949 Ext. 2763

Supplementary tables

Table S1. Primer sequences used for the second PCR in the libraries' preparation protocol. In red on the first sequence the specific barcode.

Primer 1	Sequence	Barcode
Primer1_D501	AATGATACGGCGACCACCGAGATCTACACATCGTACGACACTCTTTCCCTACACGA	ATCGTACG
Primer1_S502	AATGATACGGCGACCACCGAGATCTACACCTCTCTATACACTCTTTCCCTACACGA	CTCTCTAT
Primer1_S503	AATGATACGGCGACCACCGAGATCTACACTATCCTCTACACTCTTTCCCTACACGA	TATCCTCT
Primer1_S505	AATGATACGGCGACCACCGAGATCTACACGTAAGGAGACACTCTTTCCCTACACGA	GTAAGGAG
Primer1_S506	AATGATACGGCGACCACCGAGATCTACACACTGCATAACACTCTTTCCCTACACGA	ACTGCATA
Primer1_S507	AATGATACGGCGACCACCGAGATCTACACAAGGAGTAACACTCTTTCCCTACACGA	AAGGAGTA
Primer1_S508	AATGATACGGCGACCACCGAGATCTACACCTAAGCCTACACTCTTTCCCTACACGA	CTAAGCCT
Primer1_S510	AATGATACGGCGACCACCGAGATCTACACCGTCTAATACACTCTTTCCCTACACGA	CGTCTAAT
Primer1_S511	AATGATACGGCGACCACCGAGATCTACACTCTCTCCGACACTCTTTCCCTACACGA	TCTCTCCG
Primer1_S513	AATGATACGGCGACCACCGAGATCTACACTCGACTAGACACTCTTTCCCTACACGA	TCGACTAG
Primer1_S515	AATGATACGGCGACCACCGAGATCTACACTTCTAGCTACACTCTTTCCCTACACGA	TTCTAGCT
Primer1_S516	AATGATACGGCGACCACCGAGATCTACACCCTAGAGTACACTCTTTCCCTACACGA	CCTAGAGT
Primer1_S517	AATGATACGGCGACCACCGAGATCTACACGCGTAAGAACACTCTTTCCCTACACGA	GCGTAAGA
Primer1_S518	AATGATACGGCGACCACCGAGATCTACACCTATTAAGACACTCTTTCCCTACACGA	CTATTAAG
Primer1_S520	AATGATACGGCGACCACCGAGATCTACACAAGGCTATACACTCTTTCCCTACACGA	AAGGCTAT
Primer1_S521	AATGATACGGCGACCACCGAGATCTACACGAGCCTTAACACTCTTTCCCTACACGA	GAGCCTTA

Primer 2	Sequence	Barcode
Primer2_D701	CAAGCAGAAGACGGCATACGAGATAACTCTCGGTGACTGGAGTTCAGACGTGTG	AACTCTCG
Primer2_N701	CAAGCAGAAGACGGCATACGAGATTCGCCTTAGTGACTGGAGTTCAGACGTGTG	TCGCCTTA
Primer2_N702	CAAGCAGAAGACGGCATACGAGATCTAGTACGGTGACTGGAGTTCAGACGTGTG	CTAGTACG
Primer2_N703	CAAGCAGAAGACGGCATACGAGATTTCTGCCTGTGACTGGAGTTCAGACGTGTG	TTCTGCCT
Primer2_N704	CAAGCAGAAGACGGCATACGAGATGCTCAGGAGTGACTGGAGTTCAGACGTGTG	GCTCAGGA
Primer2_N705	CAAGCAGAAGACGGCATACGAGATAGGAGTCCGTGACTGGAGTTCAGACGTGTG	AGGAGTCC
Primer2_N706	CAAGCAGAAGACGGCATACGAGATCATGCCTAGTGACTGGAGTTCAGACGTGTG	CATGCCTA
Primer2_N707	CAAGCAGAAGACGGCATACGAGATGTAGAGAGGTGACTGGAGTTCAGACGTGTG	GTAGAGAG
Primer2_N710	CAAGCAGAAGACGGCATACGAGATCAGCCTCGGTGACTGGAGTTCAGACGTGTG	CAGCCTCG
Primer2_N711	CAAGCAGAAGACGGCATACGAGATTGCCTCTTGTGACTGGAGTTCAGACGTGTG	TGCCTCTT
Primer2_N712	CAAGCAGAAGACGGCATACGAGATTCCTCTACGTGACTGGAGTTCAGACGTGTG	TCCTCTAC
Primer2_N714	CAAGCAGAAGACGGCATACGAGATTCATGAGCGTGACTGGAGTTCAGACGTGTG	TCATGAGC
Primer2_N715	CAAGCAGAAGACGGCATACGAGATCCTGAGATGTGACTGGAGTTCAGACGTGTG	CCTGAGAT
Primer2_N716	CAAGCAGAAGACGGCATACGAGATTAGCGAGTGTGACTGGAGTTCAGACGTGTG	TAGCGAGT
Primer2_N718	CAAGCAGAAGACGGCATACGAGATGTAGCTCCGTGACTGGAGTTCAGACGTGTG	GTAGCTCC
Primer2_N719	CAAGCAGAAGACGGCATACGAGATTACTACGCGTGACTGGAGTTCAGACGTGTG	TACTACGC
Primer2_N720	CAAGCAGAAGACGGCATACGAGATAGGCTCCGGTGACTGGAGTTCAGACGTGTG	AGGCTCCG
Primer2_N721	CAAGCAGAAGACGGCATACGAGATGCAGCGTAGTGACTGGAGTTCAGACGTGTG	GCAGCGTA
Primer2_N722	CAAGCAGAAGACGGCATACGAGATCTGCGCATGTGACTGGAGTTCAGACGTGTG	CTGCGCAT
Primer2_N723	CAAGCAGAAGACGGCATACGAGATGAGCGCTAGTGACTGGAGTTCAGACGTGTG	GAGCGCTA

Table S2. Genbank accession numbers of the *16S rRNA* gene sequences from *Salmonella*, *Escherichia coli* and *Shigella* retrieved from the Refseq collection.

Accession Number	Species
NR_074910.1	Salmonella enterica subsp. enterica strain LT2
NR_074888.1	Salmonella bongori strain NCTC 12419
NR_074799.1	Salmonella enterica subsp. enterica strain Ty2
NR_116126.1	Salmonella enterica subsp. enterica serovar Typhimurium strain ATCC 13311
NR_041696.1	Salmonella enterica subsp. arizonae strain ATCC 13314
AF276989.1	Salmonella enterica
NR_119108.1	Salmonella enterica subsp. enterica strain ATCC 13311
NR_116125.1	Salmonella enterica subsp. arizonae strain DSM 9386
NR_116124.1	Salmonella bongori strain DSM 13772
NR_104709.1	Salmonella enterica subsp. enterica strain LT2
NR_044373.1	Salmonella enterica subsp. diarizonae strain DSM 14847
NR_044372.1	Salmonella enterica subsp. salamae strain DSM 9220
NR_044371.1	Salmonella enterica subsp. houtenae strain DSM 9221
NR_044370.1	Salmonella enterica subsp. indica strain DSM 14848
NR_026332.1	Shigella dysenteriae strain ATCC 13313
NR_026331.1	Shigella flexneri strain ATCC 29903
NR_104901.1	Shigella boydii strain P288
NR_104826.1	Shigella sonnei strain CECT 4887
NR_114042.1	Escherichia coli strain NBRC 102203
NR_024570.1	Escherichia coli strain U 5/41
NR_112558.1	Escherichia coli strain JCM 1649

Table S3. Genbank accession numbers of the *16S rRNA* gene sequences from *Helicobacter* species retrieved from the Refseq collection.

Accession Number	Species
NR_044761.1	Helicobacter pylori strain ATCC 43504
NR_114587.1	Helicobacter pylori strain ATCC 43504
NR_025940.1	Helicobacter acinonychis strain Eaton 90-119-3
NR_025941.1	Helicobacter cinaedi strain 165
NR_114604.2	Helicobacter fennelliae strain NCTC 11612
NR_118766.2	Helicobacter fennelliae strain Fennel 231
NR_025939.1	Helicobacter muridarum strain ST1
NR_114585.1	Helicobacter muridarum strain ATCC 49282
NR_029169.1	Helicobacter mustelae strain R85-13-6
NR_118666.1	Capnocytophaga canimorsus strain ATCC 35979
NR_025905.1	Arcobacter cryaerophilus strain A 169/B
NR_043052.1	Helicobacter canis strain ATCC 51401
NR_025952.1	Helicobacter hepaticus strain Hh-2
NR_114584.1	Helicobacter hepaticus strain ATCC 51448

NR_117866.1	Helicobacter heilmannii strain ASB1
NR_029182.3	Helicobacter bilis strain Hb1
NR_043108.1	Helicobacter cholecystus strain ATCC 700242
NR_118812.1	Helicobacter cholecystus strain Hkb-1
NR_026029.1	Helicobacter trogontum strain LRB 8581
NR_026065.1	Helicobacter salomonis strain Inkinen
NR_026074.1	Helicobacter rodentium strain MIT 95-1707
NR_024836.1	Helicobacter ganmani strain CMRI H02
NR_041748.2	Helicobacter typhlonius strain MIT 97-6810
NR_024923.1	Helicobacter mesocricetorum strain MU97-1514
NR_028019.1	Helicobacter pametensis strain B9A Seymour
NR_114586.1	Helicobacter pametensis strain ATCC 51478
NR_119304.1	Helicobacter pylori NCTC 11637 = CCUG 17874 = ATCC 43504
NR_025096.1	Helicobacter canadensis strain NLEP-16143
NR_115104.1	Helicobacter canadensis strain CCUG 47163
NR_025124.1	Helicobacter aurati strain MIT 97-5075c
NR_041825.1	Helicobacter marmotae strain MIT 98-6070
NR_114557.1	Helicobacter winghamensis strain NLEP 97-1090
NR_115314.2	Helicobacter mastomyrinus strain MIT 97-5574
NR_043457.1	Helicobacter cynogastricus strain JKM4
NR_043707.1	Helicobacter equorum strain EqF1
NR_115105.1	Helicobacter equorum strain CCUG 52199
NR_043798.1	Helicobacter anseris strain MIT 04-9362
NR_043799.1	Helicobacter brantae strain MIT 04-9366
NR_114600.3	Helicobacter macacae strain MIT 99-5501
NR_149210.1	Helicobacter japonicus strain MIT 01-6451
NR_044067.1	Helicobacter baculiformis strain M50
NR_114605.1	Helicobacter cinaedi CCUG 18818 = ATCC BAA-847
NR_043053.1	Helicobacter pullorum NCTC 12824 strain ATCC 51801
NR_114955.1	Helicobacter pullorum NCTC 12824 strain ATCC 51801
NR_115106.1	Helicobacter pullorum NCTC 12824 strain CCUG 33837
NR_116595.1	Helicobacter pullorum NCTC 12824
NR_044169.1	Helicobacter suis HS1
NR_025935.1	Helicobacter felis ATCC 49179 strain CS1
NR_026372.1	Helicobacter bizzozeronii CCUG 35545 strain CCUG 35045
NR_041808.1	Helicobacter cetorum MIT 99-5656
NR_074476.2	Helicobacter cetorum MIT 99-5656
NR_133761.1	Helicobacter valdiviensis strain WBE14
NR_151917.1	Helicobacter apri strain A19

NR_135861.1	Helicobacter himalayensis strain 80(YS1)
NR_149209.1	Helicobacter jaachi strain MIT 09-6949
NR_146694.1	Helicobacter canicola strain PAGU 1410
AY192527	Flexispira rappini strain 204-2
AY192528	Flexispira rappini strain 205-2
AY126479	Flexispira rappini strain 205-1
L12765	Flexispira rappini
