# Supplementary material for: The gut microbiota of chickens in a commercial farm treated with a Salmonella phage cocktail
Source: Sci Rep. 2022 Jan 19;12:991. doi: 10.1038/s41598-021-04679-6 (PMC8770602; doi:10.1038/s41598-021-04679-6)
Supplement: Supplementary file 3 — Supplementary Information. [file 41598_2021_4679_MOESM3_ESM.pdf]

Supplementary material

Salmonella Phage cocktail, its effects and benefits on the gut of chickens in a commercial farm.

Viviana Clavijo[a]; Tatiana Morales[b]; Martha Josefina Vives Flores[b] and Alejandro Reyes Muñoz[a,c,d*]

a. Grupo de investigación en Biología Computacional y Ecología Microbiana, Universidad de los Andes, Bogotá, Colombia. Cra 1 #18A-12, Bogotá, Colombia
b. Centro de Investigaciones Microbiológicas, Universidad de los Andes. Carrera 1 Este #19A-40, Bogotá, Colombia
c. Max Planck Tandem Group in Computational Biology, Universidad de los Andes, Carrera 1 Este #19A-40, Bogotá, Colombia
d. Center for Genome Sciences and Systems Biology, Washington University School of Medicine, Saint Louis, MO, 63108, U.S.A

* Corresponding author: a.reyes@uniandes.edu.co, Telephone: +57 1 3394949 Ext. 2763

Parameters and commands used for bioinformatic analysis

#Import data to qiime 2- PairedEndSequences

```
qiime tools import \
--type 'Sample Data[PairedEndSequencesWithQuality]' \
 --input-path /hpcfs/home/ciencias/biologia/postgrado/in-clavi/microbiome/Datos/
Datos_qiime2 \
  --input-format CasavaOneEightSingleLanePerSampleDirFmt \
  --output-path microbiome/Datos/qiime2/demux-paired-end.qza
```

#quality check

```
qiime demux summarize \
--i-data demux-paired-end.qza \
--o-visualization trimmed-seqs.qzv
```

#removing adapters using cutadapt plugin

```
qiime cutadapt trim-paired --i-demultiplexed-sequences demux-paired-end.qza --p-
anywhere-f GTGCCAGCMGCCGCGGTAA --p-anywhere-r ATTAGAWACCCBDGTAGTCC -
-p-error-rate 0 --o-trimmed-sequences trimmed-seqs.qza --verbose

 grep -c "GTGCCAGC" 95N2_S215_L001_R1_001.fastq
 grep -c "ATTCC" 95N2_S215_L001_R2_001.fastq
```

#export sequences

```
qiime tools export \
  --input-path feature-table.qza \
  --output-path exported-feature-table
```

#Feature table through Dada2

```
qiime dada2 denoise-paired   --i-demultiplexed-seqs trimmed-seqs.qza   --p-trunc-len-
f 230   --p-trunc-len-r  214   --o-representative-sequences rep-seqs-dada2.qza   --o-
table table-dada2.qza   --o-denoising-stats stats-dada2.qza
```

#Filtering samples with less than 3000 reads

```
qiime feature-table filter-samples \
  --i-table table-dada2.qza \
  --p-min-frequency 4000 \
  --o-filtered-table sample-frequency-filtered-table.qzap-cd
```

#Filtering feature table

```
qiime feature-table filter-samples --i-table table-Deblur.qza --m-metadata-file
../../metadata.tsv --p-where "Cycle='II'" --o-filtered-table table-Cycle2.qza
```

#Join feature tables

qiime feature-table merge --i-tables table-Deblur-filtered.qza --i-tables filtered_Enterobactereaceae.qza --p-overlap-method sum --o-merged-table Table_Deblur_final.qza

#visualize metadata
qiime metadata tabulate --m-input-file microbiome/Datos/qiime2/stats-dada2.qza --o-visualization microbiome/Datos/qiime2/stats-dada2.qzv

#Generating phylogenetic tree
```
qiime fragment-insertion sepp \
  --i-representative-sequences rep-seqs.qza \
  --o-tree insertion-tree.qza \
  --o-placements insertion-placements.qza
```

#Filtering Enterobacteriaceae
qiime taxa filter-table --i-table table-dada2.qza --i-taxonomy taxonomy-dada2_2.qza --p-include "D_0__Bacteria;D_1__Proteobacteria;D_2__Gammaproteobacteria;D_3__Enterobacteriales;D_4__Enterobacteriaceae" --o-filtered-table table_Enterobacteriaceae.qza

#Filtering ASVs
qiime feature-table filter-features --i-table table-Deblur-filtered_temp.qza --p-min-samples 2 --p-min-frequency 10 --o-filtered-table table-Deblur-filtered_final.qza

#Visualizing feature-table
```
qiime feature-table summarize \
  --i-table table.qza \
  --m-sample-metadata-file sample_metadata.tsv \
  --o-visualization table.qzv
```

alpha and beta diversity
qiime diversity core-metrics-phylogenetic --i-phylogeny insertion-tree.qza --i-table table.qza --m-metadata-file metadata.tsv --p-sampling-depth 4000 --output-dir core-metrics-results/

#Taxonomy
```
qiime feature-classifier classify-sklearn \
  --i-classifier gg-13-8-99-515-806-nb-classifier.qza \
  --i-reads rep-seqs.qza \
  --o-classification taxonomy.qza
```

#visualizing taxonomy
```
qiime metadata tabulate \
  --m-input-file taxonomy.qza \
  --o-visualization taxonomy.qzv
```

<u># Clustering with upgma</u>

```
module load qiime 1
upgma_cluster.py -i $PWD/beta_div.txt -o $PWD/beta_div_cluster.tre
```

<u>#To convert .biom a txt</u>

<u># Removing Salmonella ASVs</u>

```
/hpcfs/home/ciencias/biologia/docentes/a.reyes/bin/scripts/GetLinesBeginWith.pl
ToGet.txt otu_table_rdp_frombiom.txt > Filtered_otu_table.txt
```

<u>#Generation of ASVs Tree</u>

```
FastTree -nt alineamiento.fasta > tre_alineamiento.tre
```

<u># Extraction of core features</u>

```
core-features --i-table Table_time_Planta_filtered.qza --o-visualization
core_timePlanta.qzv
```

<u>#ancom analysis</u>

```
qiime taxa collapse \
  --i-table disease-table.qza \
  --i-taxonomy taxonomy.qza \
  --p-level 6 \
  --o-collapsed-table disease-table-l6.qza

qiime composition add-pseudocount \
  --i-table disease-table-l6.qza \
  --o-composition-table comp-disease-table-l6.qza

qiime composition ancom \
  --i-table comp-disease-table-l6.qza \
  --m-metadata-file sample_metadata.tsv \
  --m-metadata-column disease_state \
  --o-visualization l6-ancom-disease-state.qzvtax
```
